# Neuromuscular Plasticity Induced by Resistance Training Under Different Hypoxic Environments

**DOI:** 10.3390/brainsci16101044

**Published:** 2026-09-29

**Authors:** Gonzalo Márquez, David Colomer-Poveda, Cristina Benavente, Paulino Padial, Rafael Timón, Blanca de la Fuente, Belén Feriche

**Affiliations:** 1 Motor Control Group (ACoM), Department of Physical Education and Sport, Faculty of Sports Sciences and Physical Education, University of A Coruña, 15179 A Coruña, Spain; 2 Sport Sciences Research Centre, Faculty of Education and Sport Sciences and Interdisciplinary Studies, Rey Juan Carlos University, 28942 Madrid, Spain; david.colomerp@urjc.es; 3 Department of Physical Education and Sport, Faculty of Sport Sciences, University of Granada, 18011 Granada, Spain; cbenavente@ugr.es (C.B.); ppadial@ugr.es (P.P.); 4 Sport and Health University Research Institute (iMUDS), University of Granada, 18016 Granada, Spain; 5 Faculty of Sport Sciences, University of Extremadura, 10003 Cáceres, Spain; rtimon@unex.es; 6 High-Performance Centre of Sierra Nevada, Spanish Sports Council, 18196 Granada, Spain; blanca.delafuente@csd.gob.es

**Keywords:** strength training, hypoxia, neuromuscular adaptation, corticospinal excitability, muscle hypertrophy

## Abstract

**Highlights:**

**What are the main findings?**

- Eight weeks of hypertrophy-oriented resistance training reduced motor-evoked potentials from the recruitment curve (RC_MEPmax) after training in all conditions.
- The concomitant increase in Mmax and reduction in RC_MEPmax, alongside gains in maximal strength, suggest training-related neuromuscular adjustments enhancing motor efficiency under hypoxic stress.

**What are the implications of the main findings?**

- Moderate hypoxia did not provide clear evidence of superior neuromuscular adaptations compared with resistance training in normoxia.
- The coupled rise in Mmax and reduction in RC_MEPmax point to a coordinated peripheral–neural adaptation, opening a promising line of research into spinal excitability and motor-unit behavior across hypoxic environments.

**Abstract:**

**Background**: This study compared the effects of 8 weeks of hypertrophy-oriented resistance training (R_T_) under normoxia (N), moderate hypobaric hypoxia (HH), and moderate normobaric hypoxia (NH) on functional, neuromuscular and structural adaptations of the quadriceps. **Methods**: Twenty-nine resistance-trained males (22.5 ± 3.4 years) were assigned to N *(n* = 10), HH at 2320 m (*n* = 8), or equivalent NH (FiO_2_ = 15.9%; ~2320 m; *n* = 11). Participants completed 22 supervised R_T_ sessions across 8 weeks. Primary outcomes were corticospinal excitability (CSE), assessed via transcranial magnetic stimulation (TMS), and neuromuscular activation, assessed with femoral nerve stimulation and surface electromyography. Secondary measures included back squat one-repetition maximum (1RM_SQ) and vastus lateralis (VL) muscle thickness (MTh) via ultrasound. **Results**: All groups improved maximal squat performance (*p* < 0.001; NH Δ29.10 kg, d = −1.72; HH Δ22.61 kg, d = −1.34; N Δ16.82 kg, d = −0.99), VL MTh (*p* < 0.001; HH Δ0.25 cm, d = −0.66; N Δ0.24 cm, d = −0.63; NH Δ0.10 cm, d = −0.25), and VL maximal compound muscle action potential (Mmax) (*p* = 0.035; HH Δ1.13 mV, d = −0.65; NH Δ1.07 mV, d = −0.61; N Δ0.61 mV, d = −0.35), regardless of environmental condition. Conversely, the maximum motor-evoked potential obtained from the recruitment curve (RC_MEPmax) decreased following RT (*p* < 0.001; NH Δ−0.16, d = 1.39; HH Δ−0.08, d = 0.73; N Δ−0.04, d = 0.36). Other CSE markers remained unchanged. **Conclusions**: Eight weeks of RT under moderate hypoxia induced increases in Mmax and reductions in RC_MEPmax, suggesting training-related neuromuscular adjustments enhancing motor efficiency under hypoxic stress. These adaptations were accompanied by meaningful strength gains, although the mechanisms underlying their relationship remain to be fully elucidated.

## 1. Introduction

Exposure to hypoxic environments has emerged in recent decades as an innovative training strategy to optimize sports performance and promote specific physiological adaptations. Initially popularized in aerobic endurance disciplines, hypoxic training has the potential to stimulate additional physiological responses beyond those observed under normoxic conditions [1]. These adaptations include increases in muscle capillary density, alterations in energy metabolism, and enhanced hormonal responses, all of which may facilitate greater motor unit recruitment and, consequently, greater muscular adaptations [2,3,4,5].

From a muscle structural perspective, hypertrophy-oriented resistance training (R_T_) under hypoxic conditions may produce architectural adaptations that are similar or slightly superior to those observed in normoxia, particularly regarding muscle thickness [6,7]. These adaptations may be mediated by altered motor unit recruitment patterns resulting from increased anaerobic metabolism and the accumulation of metabolic stressors [e.g., increases in H^+^, inorganic phosphate (P_i_), reactive oxygen species (ROS)], thereby affecting key hypertrophic pathways such as anabolic hormonal responses, cell swelling, and modulation of myokines [e.g., changes in IL-6, IL-10, myostatin] [6,8,9].

From a functional perspective, chronic hypoxic training improves maximal and explosive contraction force [10], although evidence of its superiority over normoxic training remains inconsistent [6,11]. However, apart from the previously mentioned structural changes, other possible underlying mechanisms, such as subtle changes in neuromuscular transmission, spinal, cortical, and corticospinal excitability, remain poorly understood. Previous studies have shown that acute exposure to hypoxic conditions can significantly modify corticospinal excitability. For example, studies using transcranial magnetic stimulation (TMS) suggest that brief exposure to moderate altitudes temporarily increases cortical excitability [12] and enhances aspects of neuromuscular performance, such as jump height [12]. Additionally, intermittent hypoxia increases corticospinal synaptic plasticity [13], which may underlie improvements in neuromuscular function.

Hypoxia can be induced through hypobaric hypoxia (HH), achieved by exposure to moderate or high altitudes, or through normobaric hypoxia (NH), which reduces the fraction of inspired oxygen (FiO_2_) while maintaining normal barometric pressure [2]. Current research on R_T_ under hypoxic conditions has predominantly focused on NH, despite evidence that terrestrial altitude elicits distinct and often more pronounced physiological responses [14]. Although direct comparisons between R_T_ performed under NH and HH conditions are limited, recent findings suggest divergent adaptations. One comparative study using equivalent moderate FiO_2_ levels (15.9%) showed improvements in one-repetition maximum (1RM) across both conditions; however, NH revealed the most significant strength gains [15]. Paradoxically, NH resulted in a smaller muscle thickness increase, suggesting that this condition may be less effective for hypertrophy despite its positive effect on strength [16]. These findings underscore the importance of distinguishing between hypoxic environments, as R_T_ under NH and HH trigger different neuromuscular and molecular responses from the onset of exposure [2,7,8,16]. Importantly, the greater gains in strength despite similar or smaller increases in muscle size suggest that neural adaptations may have contributed to the disproportionate improvement in force-generating capacity.

Therefore, the primary objective of this study was to compare the effects of 8 weeks of hypertrophy-oriented resistance training under N, moderate HH, and moderate NH conditions on corticospinal excitability and neuromuscular activation of the quadriceps, with maximal strength and muscle thickness included as complementary functional and structural outcomes. We hypothesized that hypoxia would induce significantly greater improvements in these variables compared to training in N, primarily due to greater metabolic stress and increased neuromuscular activation. Furthermore, the type of hypoxia is expected to influence the magnitude of these adaptations.

## 2. Materials and Methods

### 2.1. Participants

Participants were part of a cohort evaluated in previous studies [15,16]. Specifically, internal and external training load variables were previously described [15], and 1RM, MTh and hormonal/myokine outcomes were reported elsewhere [16]. The present study focuses on novel neuromuscular and corticospinal excitability measures, including Mmax obtained by femoral nerve stimulation, alongside 1RM and MTh for completeness and to properly contextualize the corticospinal adaptations.

A priori sample size was determined using G*Power (v3.1, University of Kiel, Germany). For a repeated-measures design (3 groups and 2 measurement points), assuming an effect size of f = 0.3, an alpha level of 0.05, and a statistical power of 0.80, a total sample size of 30 participants was calculated. Although thirty-three strength-trained males were initially recruited and all completed the training program with complete adherence, technical issues during the neuromuscular assessments resulted in the loss of data from four participants. Therefore, twenty-nine were ultimately included in the analysis. To qualify for inclusion in the study, participants were required to meet the following inclusion criteria: (1) age between 18 and 30 years; (2) resistance-trained status, defined as consistently lifting weights at least three times per week for at least two years; and (3) sea-level residency. The exclusion criteria were (1) presence of muscular or health disorders; (2) self-reported use of performance-enhancing or anabolic agents during the previous month; (3) reported contraindications to TMS; and (4) exposure to an altitude or hypoxia environment above 1500 m asl within the two months before the study. This study was approved by the Local Research Ethics Committee (PEIBA/2018) and conducted following the Helsinki Declaration. Informed written consent was obtained from all participants before beginning the study.

### 2.2. Study Design

A longitudinal design with inter- and intra-group measurements was employed to analyze the influence of the type of moderate hypoxia (terrestrial vs. normobaric) on corticospinal excitability, strength and muscle thickness after an 8-week R_T_ program (22 sessions) with respect to the same training in normoxia. Participants were assigned to one of the three training groups using a non-randomized convenience allocation based on participants’ availability and logistical constraints associated with the different environmental training conditions. The N group lived and trained in normoxia (FiO_2_ = 20.9%; ~760 mmHg; *n* = 10, age: 22.7 ± 3.4 years; height: 175.3 ± 4.1 cm; body mass: 72.0 ± 7.7 kg), the NH group lived in N and trained in NH (FiO_2_ = 15.9%; ~760 mmHg; ~2.320 m; *n* = 11, age: 21.9 ± 2.4 years; height: 177.6 ± 7.6 cm; body mass: 76.7 ± 8.4 kg) and the HH group lived in normoxia and trained in hypobaric hypoxia (2320 m asl; ~570 mmHg; *n* = 8, age: 22.8 ± 4.3 years; height: 177.3 ± 7.8 cm; body mass: 76.2 ± 13.0 kg).

**Figure 1:**
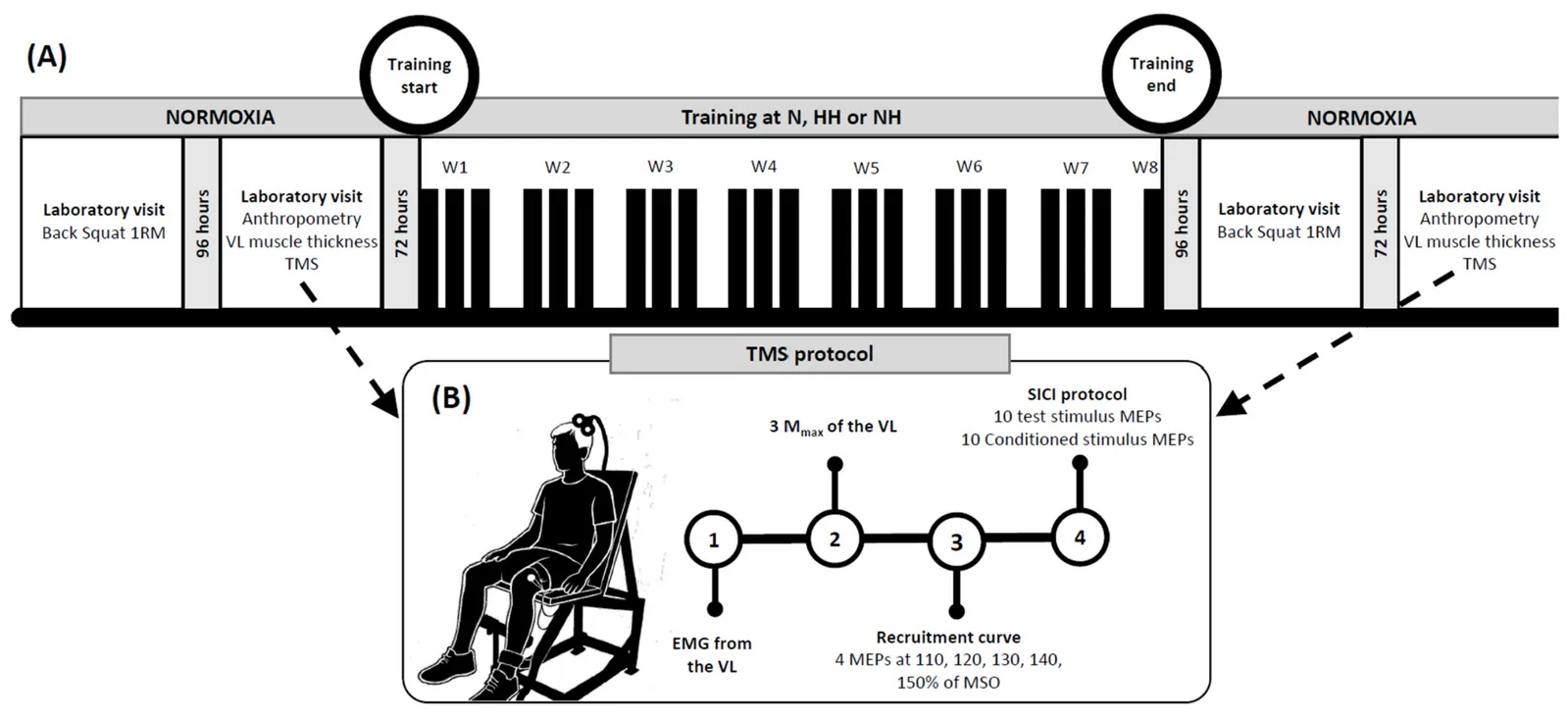
Schematic overview of the measurements obtained before and after the intervention across all groups (**A**) and the description of the TMS protocol obtained before and after the training intervention (**B**). W1 to W8: week 1 to week 8; 1RM: 1-repetition maximum; VL: vastus lateralis; TMS: transcranial magnetic stimulation; N: normoxia; HH: hypobaric hypoxia; NH: normobaric hypoxia; EMG: electromyography; Mmax: maximal compound muscle action potential; MEPs: motor evoked potential; MSO: maximal stimulator output; SICI: short intracortical inhibition.

The week before the intervention started, and 48 h after the R_T_ program, participants visited the laboratory to determine the 2–3-repetition maximum (RM) load used for 1RM estimation on the back squat exercise. Moreover, the loads used for training for the remaining exercises were determined the week before the training period. Seventy-two hours before the study and after 48 h of rest after the 1RM assessment, participants were measured for height (Seca 202, Seca Ltd., Hamburg, Germany), body mass (Tanita TBC-300, Tokyo, Japan), VL muscle thickness and neuromuscular function. An overview of the design is displayed in Figure 1.

All participants agreed to adhere to the prescribed R_T_ during the 8 weeks of the program, with no intense exercise performed other than prescribed. Participants were instructed to maintain their habits and regular dietary consumption during the entire measurement and training phases. They were provided with a protein shake supplement (111 kcal; 1.29 g of carbohydrate and 26 g of protein per serving) immediately after each session to ensure standardized nutritional intake.

All the sessions were conducted at the same time of day under the conditions of ~22 °C, ~60% humidity and <1100 ppm CO_2_ in N; ~22 °C, ~28% humidity and <1100 ppm CO_2_ in HH; and ~23 °C, 60–90% humidity and ~from 1500 to 6300 ppm CO_2_ in NH. The hypoxic environmental conditions were assessed by arterial oxygen saturation 5 min after the exposure to the assigned environmental condition (SpO_2_; Wristox 3100; Nonin, Plymouth, MN, USA). Participants’ mean resting SpO_2_ values equated to 97.2 ± 1.3, 93.9 ± 1.6 and 94.8 ± 2.2% for the first and 98.1 ± 1.5, 94.6 ± 1.3 and 94.7 ± 1.9% for the last session in N, HH and NH, respectively.

### 2.3. Hypoxic Exposure

The NH performed the training under simulated hypoxia in a normobaric tent (CAT 310, Colorado Altitude Training, Lafayette, CO, USA, 2.18 × 2.89 × 1.82 m). The hypoxic generator system pumped the air through a semi-permeable filtration membrane (nitrogen filter technique; CAT 12, Colorado Altitude Training, Lafayette, CO, USA, 100 L/min), depleting the oxygen content until reaching an FiO_2_ = 15.9%, according to the manufacturer’s guidelines to equate an altitude of 2320 m. Ambient O_2_ was continuously monitored by a digital controller (Handi+, Maxtec, Salt Lake City, UT, USA) to maintain the hypoxic conditions in the tent. Two participants trained simultaneously in the tent. Consistent with conventional routine [17,18], NH participants entered the tent and sat for 5 min to adapt to the training environment before starting their warm-up. The HH group performed the training sessions under terrestrial HH conditions at the High-Performance Center of Sierra Nevada (2320 m asl., Spain). Participants remained at altitude only during the training sessions.

### 2.4. Resistance Training Program

The 8-week R_T_ program consisted of three sessions per week performed on non-consecutive days, plus an extra rest day at the end of the week [16]. Training sessions comprised a full-body routine of 6 exercises (knee-dominant lower-body, hip-dominant lower-body, upper-body vertical and horizontal push, and upper-body vertical and horizontal pull), each performed for three sets of 6–12 repetitions [19], with a load ranging from 65 to 80% of 1RM and 90 s of rest between sets and exercises [20]. The training load and volume fluctuated throughout the week (i.e., 10 repetitions per set at 70% 1RM was used in each first session of each week, 6 repetitions per set at 80% 1RM in the second and 12 repetitions per set at 65% 1RM in the third). The detailed exercise progression and daily exercise selection have been previously published in full [15]. The load was individually adjusted by ~5% when participants exceeded the target repetition range while using a proper technique, in accordance with conventional methods [21]. The same experts directly supervised all routines to guarantee correct technique and safe execution. Consistent encouragement was provided for participants throughout all training sessions.

### 2.5. One-Repetition Maximum

Each participant’s 1RM was calculated for the back squat according to the National Strength and Conditioning Association guidelines [22]. Prior to testing, participants performed a warm-up consisting of cardiovascular activation on a cycle ergometer lasting 5–10 min, followed by a set of 5 repetitions at ~50% of their estimated 1RM and after, 1–2 sets more of 2–3 repetitions at a load corresponding to ~60–80% of the estimated 1RM for the exercise. Three sets of 3–6 repetitions at increasing loads were completed before performing 1 set of 2–3 repetitions to failure. The 2–3RM load was used for 1RM estimation from the validated Brzycki’s equation [23]. Between each successive attempt, participants rested for 5 min. All 1RM determinations were made within 3 attempts. After the R_T_ program, participants repeated the same 1RM assessment for the back squat as an indicator of strength improvement from the interventions.

### 2.6. Muscle Thickness

Individual muscle thickness was measured on the dominant leg using ultrasound equipment (GE-LOGICQ-E portable model; GE Healthcare, Little Chalfont, UK) before and after the training period. The maximum thickness of the VL was obtained at 50% of the distance from the superior and middle tip of the patella to the anterior superior iliac spine [24]. The lateral location of the VL measurement was taken at 10% of the thigh circumference in the lateral direction. With the participant lying supine, the ultrasound probe (12 L linear probe at 10 MHz frequency, gain 80 dB, depth 8 cm) was oriented perpendicular to the muscle fascicles and the skin, with sufficient ultrasound gel to reduce muscle compression. The depth of the image was adjusted until the femur and muscle boundaries were visible on the screen. Three images were taken and saved for subsequent analysis. The average of the measures from three images was used for analysis. The thickness of the VL was defined as the distance from the subcutaneous adipose tissue-muscle interface to either the aponeurosis or the muscle-bone interface. The same expert performed all ultrasound measurements (CV < 1.8%) and was blinded to the condition to which each participant was assigned. This level of repeatability is consistent with previous studies reporting excellent intra-rater reliability for ultrasound measurements of vastus lateralis muscle thickness (ICC = 0.928–0.961) [25].

### 2.7. Neuromuscular Function Assessment

Neuromuscular function, comprising corticospinal excitability (CSE) and intracortical inhibition (SICI) assessed by TMS, and muscle activation, assessed by femoral nerve stimulation and surface EMG, constituted the primary outcomes of this study and were assessed before and after 8 weeks of RT in different environmental conditions, as previously mentioned. For this purpose, participants sat in a custom-made chair with the hip, knee, and ankle at 90°, and the torso restrained with belts to prevent displacement. The dominant leg was strapped just above the malleoli. Surface EMG was recorded from the VL using Ag-AgCl surface electrodes (~2 cm inter-electrode distance) attached to the skin according to SENIAM recommendations [26]. EMG signals were amplified (×250), band-pass-filtered (10–500 Hz), and sampled (2 kHz) with a Digitimer d440-isolated amplifier (Digitimer Ltd., Welwyn Garden City, Hertfordshire, UK). The EMG recordings were collected using an analog-digital board (CED Micro1401-3, Signal v6, Cambridge Electronic Design Ltd., Cambridge, UK) for further analysis.

Single-pulse TMS was delivered to the contralateral motor cortex using a concave double-cone coil (DuoMAG 120BFV Coil) which induced a posterior–anterior intracranial current connected to a DuoMag magnetic stimulator (Rogue Resolutions Ltd.; Cardiff, UK). The optimal stimulation point of each leg was obtained by exploring the estimated center of the VL cortical representation. The point at which MEP was the largest in each session was marked on the scalp with a permanent marker. The optimal stimulation site was identified independently at each testing session (pre and post) using the same functional search procedure (i.e., the scalp location eliciting the largest MEP for a given stimulation intensity), rather than relying on the scalp marking from the previous session. This functional relocalization procedure is the same one used in our group’s previous work, in which intersession reliability of Mmax- and MEP-related outcomes obtained across independent testing sessions was consistently good to excellent (ICC = 0.65–0.98) [27,28,29], supporting that re-identifying the hotspot at each session does not introduce material measurement instability. Active motor threshold (AMT) for each leg was determined as the lowest stimulation intensity that produced three out of five MEPs of a peak-to-peak amplitude >200 µV during a 5% unilateral maximum voluntary isometric contraction (MVIC), whereby the rectified EMG associated with this level was displayed as a line on a monitor in front of the participant. To measure CSE, a recruitment curve (RC) was assessed during a unilateral contraction corresponding to the same sEMG value (5% of the MVIC) used in the AMT determination. Stimulation intensity started with a subthreshold intensity of 30% of the stimulator output and increased in steps of 10% until it reached 90% of the stimulator output. Four pulses were given at each stimulation intensity. The peak-to-peak amplitude of MEPs obtained in the VL was measured offline and used to calculate the total area under the recruitment curve (AURC) using the trapezoidal integration method and the maximum MEP (average of 4 MEPs evoked at an intensity of 90% MSO) of the recruitment curve (RC_MEP_max_) as previously described [12,27]. Furthermore, to measure short intracortical inhibition (SICI), a paired-pulse protocol was used in which the intensity of the conditioning stimulus was set at 80% of the AMT, while the intensity for the test stimulus, delivered 2.5 ms later, was set at 130% of the AMT. CSE was also computed by averaging the peak-to-peak amplitude of ten unconditioned single-pulse MEPs (MEP_130%_). SICI was quantified by dividing the average of ten paired-pulse MEPs by the averaged single-pulse MEPs. The root mean square of the EMG (rmsEMG) during the 150 ms before every pulse was also measured and averaged for each session to ensure identical background EMG conditions.

The maximal compound muscle action potential (M_max_) of the VL muscle was obtained via single-pulse electrical stimulation (200 µs duration) delivered to the femoral nerve with a DS7AH constant current electrical stimulator (DS7AH, Digitimer Ltd., Welwyn Garden City, Hertfordshire, UK). The cathode (Globus motor point pen; Globus Ltd., Codognè, Italy) was located over the femoral triangle, and the anode (5 × 10 cm carbon rubber electrodes coated with electrolytic gel) was midway between the greater trochanter and the iliac crest (i.e., gluteal fossa). The intensity for stimulation was set at 130% of the stimulation intensity needed to elicit a maximum VL M_max_. Three pulses were obtained at the beginning of each session during a contraction of 5% of MVIC force, as previously described. The peak-to-peak amplitude of the M_max_ was measured and averaged. The average M_max_ value of each testing session was then used for normalization procedures of all the other MEP and EMG variables. Although a dedicated reliability analysis was not performed in the present sample, our group has previously reported good-to-excellent reliability for directly comparable measures obtained with the same equipment and protocol: intersession reliability of AMT, Mmax and the area under the recruitment curve in the knee extensors, including the vastus lateralis, ranged from good to excellent (ICC = 0.65–0.94) [27]; and, in the elbow flexors, intrasession and intersession reliability of Mmax and MEPs was good to excellent (ICC = 0.88–0.99 and 0.67–0.98, respectively) [29], with intersession reliability of Mmax, MEPs and SICI ranging from good to excellent (ICC = 0.79–0.94) [28].

### 2.8. Statistical Analysis

Prior to conducting the statistical analyses, assumptions of data normality were tested using the Shapiro–Wilk test (all *p* > 0.05). We employed the three-sigma rule of thumb method to identify outliers in the raw data [30]. Baseline comparability across the three groups (N, HH, NH) for age, height, body mass, and BMI was assessed using one-way ANOVAs, followed by Bonferroni-corrected post hoc comparisons where applicable. A two-way repeated measures analysis of variance (RM-ANOVA) was performed to assess the effects of time (pre and post) and training condition (N, HH and NH) on neuromuscular, structural and functional variables. When significant main effects or interactions were found in RM-ANOVA, post hoc comparisons were performed using Bonferroni correction. CSE and neuromuscular activation (AMT, AURC, RC_MEPmax, MEP130%, SICI and Mmax) were treated as a single, pre-specified primary outcome family, and 1RM_SQ and VL_MTh were secondary outcomes; no additional family-wise correction was applied across these eight pre-specified outcomes beyond the Bonferroni correction applied within each variable’s own post hoc comparisons. Effect sizes [ES] are presented as partial eta squared values (η_p_^2^; small: 0.01; medium: 0.06; large: 0.14) for the RM-ANOVA and as Cohen’s d for the paired comparisons. Threshold classifications for Cohen’s d were set as follows: >0.2 [small], >0.6 [moderate], >1.2 [large] and >2 [very large]. Note that these thresholds follow the modified classification proposed by Hopkins et al. [31]. Unless indicated otherwise, data are reported as mean ± standard deviation (SD). All analyses were performed using the JASP software (Version 0.19.3). Statistical significance was set at *p* ≤ 0.05.

## 3. Results

The present study investigated the effects of an 8-week hypertrophy-oriented resistance training program performed under different environmental conditions (N, NH and HH) on neuromuscular, structural and functional adaptations. Descriptive statistics (mean ± SD), within-group effect sizes (Cohen’s d), and RM-ANOVA outcomes (main effects of time and time × group interactions) are presented in Table 1.

**Figure 2:**
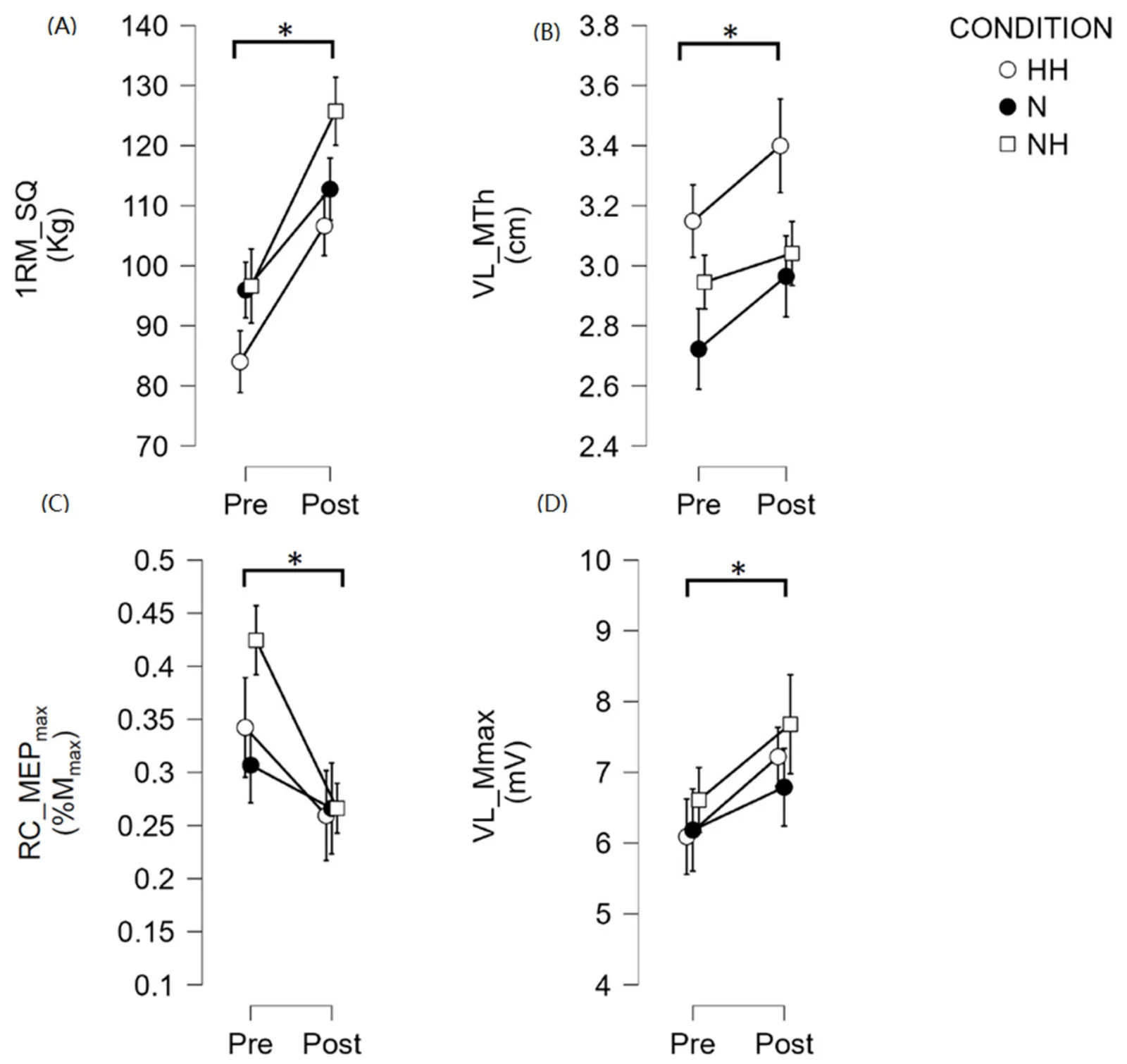
Average responses across experimental groups for (**A**) 1RM_SQ, (**B**) VL_MTh, (**C**) RC_MEP_max_ and (**D**) VL_M_max_ before (PRE) and after (POST) eight weeks of RT. Data are presented as mean ± standard error. * *p* < 0.05.

No significant baseline differences were observed between groups for any of the analyzed variables, indicating that all groups started the intervention under comparable experimental conditions. Maximum squat strength (1RM_SQ; Figure 2A) significantly increased following training (main effect of time, *p* < 0.001). A significant time × group interaction was observed (*p* = 0.019); however, Bonferroni-adjusted pairwise comparisons did not reveal significant differences in strength gains between environmental conditions (NH Δ29.10 kg, d = −1.72; HH Δ22.61 kg, d = −1.34; N Δ16.82 kg, d = −0.99). Muscle thickness (VL_MTh; Figure 2B) significantly increased after training (main effect of time, *p* < 0.001), whereas neither the main effect of condition nor the time × group interaction reached statistical significance (HH, Δ0.25 cm, d = −0.66; N Δ0.24 cm, d = −0.63; NH Δ0.10 cm, d = −0.25). Maximum motor-evoked potential amplitude obtained from the recruitment curve (RC_MEPmax; Figure 2C) significantly decreased after training (main effect of time, *p* < 0.001; NH Δ−0.16, d = 1.39; HH Δ−0.08, d = 0.73; N Δ−0.04, d = 0.36), but no significant between-condition differences were detected. Maximum M-wave amplitude (Mmax; Figure 2D) significantly increased following training (main effect of time, *p* = 0.035), with no evidence of differential responses between environmental conditions (HH Δ1.13 mV, d = −0.65; NH Δ1.07 mV, d = −0.61; N Δ0.61 mV, d = −0.35). Individual participant trajectories (pre–post) for these four variables (1RM_SQ, VL_MTh, RC_MEPmax and VL_Mmax), alongside the group means, are provided in Supplementary Figure S1.

No significant changes were observed in AMT, AURC, MEP130%, or SICI. Although none of these comparisons reached statistical significance, some descriptive trends are noteworthy: AURC decreased moderately in both hypoxic conditions (HH d = 0.67; NH d = 0.60) while remaining virtually unchanged in N (d = −0.13); MEP130% followed a similar pattern, decreasing more in HH and NH (d = 0.41 and 0.64, respectively) than in N (d = 0.09); SICI decreased slightly, denoting a small increase in intracortical inhibition, in HH and N (d = 0.43 and 0.55) but remained stable in NH (d = −0.05); and AMT showed negligible and inconsistent within-group changes (d range: −0.33 to 0.04).

## 4. Discussion

Despite growing interest in hypoxic resistance training, the extent to which moderate hypobaric and normobaric hypoxia modulate corticospinal excitability and neuromuscular activation during a hypertrophy-oriented program remains poorly understood, as most previous work has focused on structural or hormonal outcomes rather than neurophysiological ones. Clarifying whether these two hypoxic modalities elicit distinct neural adaptations is clinically and practically relevant for prescribing hypoxia-based resistance training. The present study therefore aimed to determine whether 8 weeks of hypertrophy-oriented resistance training under normoxia, moderate hypobaric hypoxia, and moderate normobaric hypoxia differentially affect corticospinal excitability and neuromuscular activation, with maximal strength and muscle thickness assessed as complementary outcomes.

In partial agreement with our hypothesis, the present findings demonstrated that eight weeks of hypertrophy-oriented resistance training elicited substantial improvements in maximal strength irrespective of the environmental condition. Although a significant time × group interaction was observed for 1RM, post hoc comparisons did not confirm statistically significant differences between N, HH and NH. Overall, these findings are consistent with previous literature indicating that resistance training performed under moderate hypoxia can promote strength adaptations comparable to, and in some cases greater than, those achieved under normoxic conditions [2,16,32,33]. Furthermore, R_T_ induced neuromuscular adaptations, such as an increase in M_max_ and a reduction in the RC_MEP_max_, regardless of the environmental condition. The descriptive divergence between strength gains and hypertrophic adaptations, particularly under NH, raises the possibility that neural mechanisms may have contributed to the observed functional improvements. However, the present neurophysiological outcomes do not allow definitive identification of the specific mechanisms involved.

### 4.1. Muscular Strength and Hypertrophy Adaptations

As hypothesized, 8 weeks of R_T_ improved maximal strength across all conditions, with greater descriptive improvements observed under hypoxic environments. This aligns with recent meta-analyses reporting only modest benefits of hypoxia on strength and muscle mass development compared to normoxia [7,34,35]. These descriptive differences may be partially explained by the specific configuration of the training program, including factors such as training intensity, recovery intervals, hypoxic severity and exercise selection, all of which have previously been identified as potential modulators of the adaptive response to resistance training performed under hypoxia [7,36]. Among the hypoxic conditions, NH exhibited the largest descriptive increase in maximal strength. However, because pairwise comparisons did not identify statistically significant differences between groups, this observation should be interpreted as a trend rather than evidence of superiority. Interestingly, muscle thickness (MTh), a marker of hypertrophic adaptations, showed small to moderate increases across all conditions. Although the descriptive increase in muscle thickness was smaller in NH, hypertrophic adaptations were observed in all three groups, and neither the main effect of condition nor the interaction reached statistical significance.

The reduction in muscle growth after R_T_ in hypoxia may be explained by the increase in internal load and metabolic stress [37,38]. This stress response may be more pronounced under HH than under NH, making HH a more physiologically demanding condition [14]. Indeed, the more reduced resting SpO_2_ in HH compared with NH at the beginning of the training program (93.9% vs. 94.8%, respectively) supports an independent effect of barometric pressure, which may induce distinct physiological responses. However, when external workload is matched, the internal responses do not significantly differ between NH and HH, suggesting that the type of hypoxia may not differentially modulate internal load under standardized training intensities [15]. Another possible explanation may be attributed to air quality factors associated with common features of NH tent systems. These systems are typically small and operate with reduced oxygen flow rates, conditions that are consistent with the elevated relative humidity and carbon dioxide levels observed inside the tent in the NH group. Augmented carbon dioxide levels reduce alveolar oxygen partial pressure regardless of any changes in the FiO_2_ [39], thereby reducing SpO_2_ beyond the level expected for a given degree of hypoxic severity [40]. This additional physiological stress could influence intracellular signaling pathways associated with muscle remodeling, potentially favoring mechanisms involved in cellular differentiation over proliferation, although this interpretation remains speculative [16,41]. Indeed, in addition to the hypoxia-induced metabolic stress on ROS and inflammatory cytokines [9], recent evidence has shown that the type of hypoxia also modulates hormonal [8,42] and myokine/miRNA responses to R_T_ linked to mechanisms underlying hypertrophic adaptations [16,41]. Finally, chronic or excessive inflammatory signaling can impair muscle regeneration and promote protein degradation [43]. Yet, although chronic inflammation may impair muscle regeneration, current evidence regarding the inflammatory response to resistance training under different hypoxic conditions remains limited, and available data suggest a proportionate response [44].

### 4.2. Neuromuscular Plasticity After RT in Different Environmental Conditions

We hypothesized that resistance training performed under hypoxic conditions would induce greater neuromuscular adaptations than training performed in normoxia. However, this hypothesis was only partially supported by the present findings. No significant changes were observed in most corticospinal excitability markers (MEP130%, AURC and SICI), whereas RC_MEPmax decreased after the intervention irrespective of the environmental condition. Importantly, no significant between-condition differences were detected for any of these neurophysiological variables. These findings appear to differ from those reported in the recent meta-analysis by Siddique et al. [45], which suggested that resistance training generally increased corticospinal excitability and reduced intracortical inhibition. However, several recent experimental studies have reported either unchanged or reduced corticospinal excitability following resistance training [27,46,47,48], indicating that increases in corticospinal excitability are unlikely to represent a universal neural adaptation to strength training.

One possible interpretation of the reduction in RC_MEPmax is that resistance training increased the force-generating capacity of the recruited motor units, thereby reducing the corticospinal output required to produce the target contraction [27,28,29,48]. Although twitch contractile properties were not assessed, the concomitant improvements in maximal strength and muscle thickness are compatible with this interpretation. Nevertheless, the present study was not designed to directly test this mechanism. An alternative, although equally speculative, explanation is that repeated resistance training enhanced the efficiency of corticospinal transmission, reducing the neural drive required to achieve the same submaximal force output. Such an adaptation could reflect a redistribution of neural contributions across cortical, subcortical and spinal levels rather than a simple reduction in corticospinal excitability [27,46,49,50]. Although the reduction in RC_MEPmax was descriptively greater following hypoxic training, particularly under NH, these differences were not statistically significant between environmental conditions. Therefore, the present findings do not provide direct evidence that moderate hypoxia induces greater corticospinal adaptations than normoxic resistance training. Nevertheless, the descriptive pattern observed warrants further investigation in adequately powered studies.

Experimental evidence indicates that both acute normobaric hypoxia and R_T_ performed under normoxic conditions may reduce GABA-B-mediated intracortical inhibition [51]. It is therefore physiologically plausible that combining R_T_ with acute hypoxia could produce a sustained reduction in intracortical inhibition. However, intracortical inhibition remained unchanged in all groups in the present study. These findings suggest that, under the conditions examined, R_T_ performed in N, HH or NH does not induce detectable changes in intracortical inhibition, which is consistent with recent evidence showing no changes in intracortical inhibition of the VL cortical representation following four weeks of R_T_ [47].

Regarding the M_max_, our results showed an increase with R_T_ across all conditions. These results agree with longitudinal findings showing M_max_ enlargement after R_T_ in hypoxia [6] and with cross-sectional studies reporting greater M_max_ amplitudes in resistance-trained versus untrained men [52]. The M-wave primarily reflects peripheral membrane excitability and the propagation of action potentials along the muscle fibers [53]. Consequently, changes in Mmax should not be interpreted as a direct measure of voluntary neural activation. In the present study, the increase in Mmax was more likely related to peripheral adaptations associated with resistance training, including increases in muscle fiber size and changes in muscle electrical properties, both of which can increase the recorded compound muscle action potential [53]. Interestingly, descriptively larger increases in Mmax were observed following HH and NH than after normoxic training. However, because no significant interaction was detected, these observations should not be interpreted as evidence of a hypoxia-specific adaptation. An alternative explanation may involve differences in peripheral tissue characteristics, such as reductions in subcutaneous adipose tissue, which could modify the muscle–electrode distance and consequently influence the recorded M-wave amplitude. Previous investigations reporting greater reductions in fat mass following resistance training under hypoxic conditions provide indirect support for this possibility [9], although this mechanism was not directly assessed in the present study.

### 4.3. Limitations

Several limitations must be considered when interpreting the results. First, the sample size was relatively small, potentially limiting statistical power and generalizability, so the interaction should be interpreted cautiously; nevertheless, the sample is of interest and aligns with those used in similar studies within this research area. Second, group allocation was conducted using a non-randomized convenience approach, which can introduce selection bias. This limitation should be considered when interpreting the findings. Third, only a single muscle group and exercise modality were examined, possibly limiting the scope of neuromuscular adaptations observed. Future research should consider longer training durations, larger and diverse participant populations (e.g., females, elderly people, etc.), different muscle groups, and comprehensive assessments of other neuromuscular (e.g., motor unit behavior) and molecular responses to further clarify the mechanisms underlying functional adaptations to R_T_ under different hypoxic conditions (i.e., HH vs. NH). Fourth, a dedicated test–retest repeatability analysis of the TMS-derived and Mmax measures was not performed in the present sample; nevertheless, directly comparable measures obtained by our group with the same equipment and an essentially identical protocol, including recordings from the vastus lateralis, have previously shown good-to-excellent intersession reliability (ICC = 0.65–0.98) [27,28,29], which supports the robustness of the present neurophysiological outcomes.

## 5. Conclusions

The present study demonstrated that eight weeks of hypertrophy-oriented resistance training performed under normoxic, normobaric hypoxic and hypobaric hypoxic conditions effectively improved maximal strength, muscle thickness and selected neuromuscular outcomes in resistance-trained individuals. Although descriptively greater strength gains and larger reductions in RC_MEPmax were observed following hypoxic training, particularly under NH, these differences were not confirmed by post hoc comparisons between environmental conditions. Overall, the observed adaptations appeared to vary according to the hypoxic modality and outcome assessed. Therefore, the present findings do not provide conclusive evidence that moderate hypoxia enhances functional or neurophysiological adaptations beyond those achieved with equivalent resistance training in normoxia. The concomitant increase in Mmax and reduction in RC_MEPmax suggest that resistance training induced both peripheral and neural adaptations that may enhance motor efficiency under hypoxic stress. However, the relative contribution of corticospinal, spinal and peripheral mechanisms to these responses remains unclear and warrants further investigation. Collectively, these findings indicate that resistance training under moderate hypoxic conditions does not provide evidence of superiority over conventional normoxic training and instead represents a comparable training strategy under the conditions examined. Future studies incorporating direct measures of spinal excitability, motor-unit behavior and peripheral muscle adaptations will be essential to clarify the physiological mechanisms underlying the potential differences observed between environmental conditions.

## Figures and Tables

**Table 1:** Pre- and post-intervention values for maximal strength (1RM_SQ), VL muscle thickness (VL_MTh), maximal compound muscle action potential of VL muscle (VL_M_max_), active motor threshold (AMT), maximal motor evoked potential from the recruitment curve (RC_MEP_max_), area under the recruitment curve (AURC), MEP amplitude at 130% of AMT (MEP_130%_), and short-interval intracortical inhibition (SICI) across the HH, NH and N groups.

		PRE		POST			TIME			TIME × GROUP		
		MEAN	SD	MEAN	SD	Cohen’s d	F	*p*	η_p_^2^	F	*p*	η_p_^2^
**1RM_SQ** (Kg)	**HH**	84.02	14.50	106.63	14.00	−1.337	174.386	**<0.001**	0.870	4.650	**0.019**	0.264
	**NH**	96.63	20.41	125.73	18.75	−1.721						
	**N**	95.94	14.67	112.78	16.38	−0.994						
**VL_MTh** (cm)	**HH**	3.15	0.34	3.40	0.44	−0.657	26.570	**<0.001**	0.505	1.879	0.173	0.126
	**NH**	2.95	0.30	3.04	0.35	−0.250						
	**N**	2.72	0.42	2.97	0.43	−0.634						
**VL_M_max_** (mV)	**HH**	6.09	1.51	7.22	1.17	−0.645	4.972	**0.035**	0.161	0.159	0.854	0.012
	**NH**	6.61	1.52	7.68	2.32	−0.611						
	**N**	6.19	1.84	6.79	1.73	−0.346						
**AMT** (%MSO)	**HH**	45.50	6.63	45.25	7.80	0.036	2.196	0.150	0.078	1.459	0.251	0.101
	**NH**	38.82	7.77	41.09	8.12	−0.326						
	**N**	37.90	5.22	38.60	5.60	−0.100						
**RC_MEP_max_** (%M_max_)	**HH**	0.34	0.13	0.26	0.12	0.726	19.410	**<0.001**	0.427	2.870	0.075	0.181
	**NH**	0.42	0.11	0.27	0.08	1.385						
	**N**	0.31	0.11	0.27	0.14	0.357						
**AURC** (a.u.)	**HH**	559.89	257.89	424.50	157.35	0.671	2.264	0.144	0.080	1.059	0.362	0.075
	**NH**	632.81	264.30	512.70	174.00	0.595						
	**N**	414.13	146.44	440.90	177.04	−0.133						
**MEP_130%_** (%Mmax)	**HH**	0.23	0.08	0.18	0.10	0.412	2.560	0.122	0.090	0.499	0.613	0.037
	**NH**	0.30	0.15	0.23	0.08	0.635						
	**N**	0.22	0.12	0.21	0.15	0.087						
**SICI** (%test_MEP)	**HH**	0.60	0.16	0.54	0.13	0.425	2.313	0.140	0.082	0.912	0.414	0.066
	**NH**	0.66	0.15	0.67	0.16	−0.054						
	**N**	0.70	0.17	0.62	0.17	0.547						

Cohen’s d indicates effect sizes for within-group changes. Results of RM-ANOVA are shown for the main effect of time and the time × group interaction, including F-values, *p*-values, and partial eta squared (η_p_^2^).

## Data Availability

The datasets generated and analyzed during the current study are available from the corresponding author upon reasonable request due to the fact that several datasets are currently being analyzed and prepared for future publications.

## Supplementary Materials

The following supporting information can be downloaded at: https://www.mdpi.com/article/10.3390/brainsci16101044/s1, Figure S1. Individual participant trajectories (Pre-Post) by condition, with group mean ± SE overlaid.
